# Chromosomal Study of Couples with the History of Recurrent Spontaneous Abortions with Diagnosed Blightded Ovum

**Published:** 2013

**Authors:** Sahar Shekoohi, Majid Mojarrad, Reza Raoofian, Shahab Ahmadzadeh, Salmah Mirzaie, Mohammad Hassanzadeh-Nazarabadi

**Affiliations:** 1 *Department of Medical Genetics, School of Medicine, Mashhad University of Medical Sciences, Mashhad, Iran.*

**Keywords:** Blighted ovum, chromosomal rearrangement, consanguinity

## Abstract

Spontaneous abortion (SAb) is the most common complication of early pregnancy. Numerous risk factors are associated with an increased risk of pregnancy loss such as: Blighted ovum. The aim of this study was to determine the frequency of balanced chromosomal translocations in couples with a history of recurrent spontaneous abortions and ultrasound diagnosed blighted ovum. Sixty Eight couples with the history of spontaneous abortion (diagnosed blighted ovum) were selected and introduced into this survey during 2007-2012 at Medical Genetics department of Mashhad University of Medical Sciences. Giemsa banding technique was used to search for chromosomal balanced translocations. Demographic assessment has not shown any age difference between blighted ovum suffering couples and general population. Consanguineous marriages in blighted ovum suffering couples was significantly higher (P *value *<0.001) than non-consanguineous marriages (68.5% versus 31.5%), while in general population 62% of were non-consanguineous. The incidences of balanced chromosomal rearrangements as well as the rate of chromosome 9 inversion were 8.3 percent each, in non-consanguineous Blighted ovum suffering couples and the remaining (83.4%) showed normal karyotypes. There was no chromosome 9 inversion in consanguineous blighted ovum suffering couples and the incidence of balanced chromosomal rearrangements was 2.3%. With regard to relatively low incidence of balanced chromosomal rearrangements in consanguineous couples with blighted ovum, it would be reasonable to suggest that single gene determinants may play an important role in such pregnancy complications rather than chromosomal disorders.

Spontaneous abortion (SAb), also known as miscarriage, refers to a pregnancy that ends spontaneously before the fetus has reached a viable gestational age (1). SAb is the most common complication of early pregnancy (1). The frequency of SAb decreases with increasing gestational age. 8 to 20 percent of clinically recognized pregnancies under 20 weeks of gestation will undergo SAb; 80 percent of these occur in the first 12 weeks of gestation (2-3).

Loss of unrecognized or subclinical preg-nancies is even higher, occurring in 13 to 26 percent of all pregnancies (4). Numerous risk factors are associated with an increased risk of pregnancy loss:

Age: Advancing maternal age is one of the risk factors for spontaneous miscarriage in healthy women (5). The overall rate of SAb was 11 percent and the approximate frequencies of clinically recognized miscarriage according to maternal age were: age 20 to 30 years (9 to 17 percent), age 35 (20 percent), age 40 (40 percent), and age 45 (80 percent) (6). Previous spontaneous abortion: Past obstetrical history is an important predictor of subsequent pregnancy outcome. The risk of miscarriage in future pregnancy is approximately 24 percent after one miscarriage, 30 percent after two consecutive miscarriages, and 35 percent after three or more consecutive miscarriages (7-8). By comparison, miscarriage occurred in only 5-10 percent of women in their first pregnancy or in whom the previous pregnancy was successful (7). Gravidity: Some studies have shown an increased risk of miscarriage with increasing gravidity. Possible reasons for this association include reproductive compensation behavior (pregnancy failure is likely to be associated with repeated attempts at conception resulting in higher gravidity) and short interpregnancy intervals in multigrain (9-10). Prolonged ovulation to implantation: some studies revealed that prolonged interval (i.e, >10 days) between ovulation and implantation may lead to early fetal loss. Fertilization of older ovum, delayed tubal transport or abnormal uterine receptivity can result to such delays (11-12). Interval Prolonged time to pregnancy: according to some observational studies, the risk of miscarriage can be increased by prolonged time to achieving pregnancy (13). Maternal weight: Prepregnancy body mass index less than 18.5 or above 25 kg/m^2^ has been associated with an increased risk of infertility and SAb (14-15). Blighted ovum: A blighted ovum is characterized through ultrasound examination by the absence of an embryo in the gestational sac (an embryonic pregnancy) (16).

One-third of the products of conception from spontaneous abortions occurring at or before 8 weeks of gestation are "blighted" or anembryonic. Blighted or otherwise abnormal embryos may result from chromosomal abnormalities or possible exposure to teratogenes.

Most chromosomal abnormalities in the embryo occur de novo. Rarely, these defects are inherited as a consequence of parental karyotype abnormalities, such as balanced translocations. Genetic abnormalities will not be detected by conventional cytogenetic analysis (G-banded karyotype) and account for an undefined proportion of spontaneous abortions (17). The aim of this study was to determine the frequency of balanced chromosomal translocations in couples with a history of recurrent spontaneous abortions who were referred to medical cytogenetic laboratory by ultrasound diagnosed blighted ovum.

## Materials and Methods

**Fig. 1:**
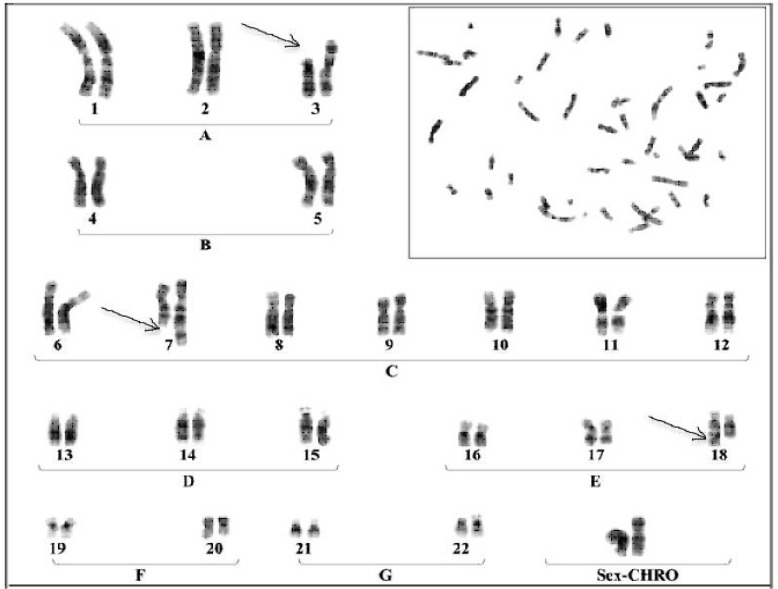
Chromosomal analysis of a female showing RSA

In order to fulfill this study, all couples referred to the cytogenetic laboratory of Mashhad University of Medical Sciences during 2007-2012 were chosen for this study after having ruled out immunologic, hormonal and anatomic factors. Finally, 68 couples with the history of spontaneous abortion (two or three consecutive miscarriages) with first trimester gestational age and blighted ovum were considered for the study. Chromosomal investigation was performed in order to detect possible balanced chromosomal rearrangements. After obtaining informed consent, five ml of peripheral whole blood were collected into a sterile heparinized tube and cultured for 3 days in RPMI 1640 medium, supplemented with 20% fetal calf serum, 50U/ml penicillin and 100 μg/ml streptomycin. The lymphocytes proliferation was stimulated by adding phytohemaglutinin. The cells were harvested by adding colchicine 2 h before slide preparation. In order to prepare slides, cells were exposed to hypotonic solution (0.05 M KCI) and then treated with trypsin. Giemsa banding was performed following fixation with 1:3 ethanol-glacial acetic acid solutions. 30-40 metaphase cells were analyzed microscopically for their chromo-some constitution. Each chromosome was analyzed for its presence and structure. Pairing 46 human chromosomes in well defined order was performed based on characteristic band pattern. The 3 pairs differ in the length of their arms and each shows a unique banding pattern (Fig. 1).

## Results

**Fig. 2:**
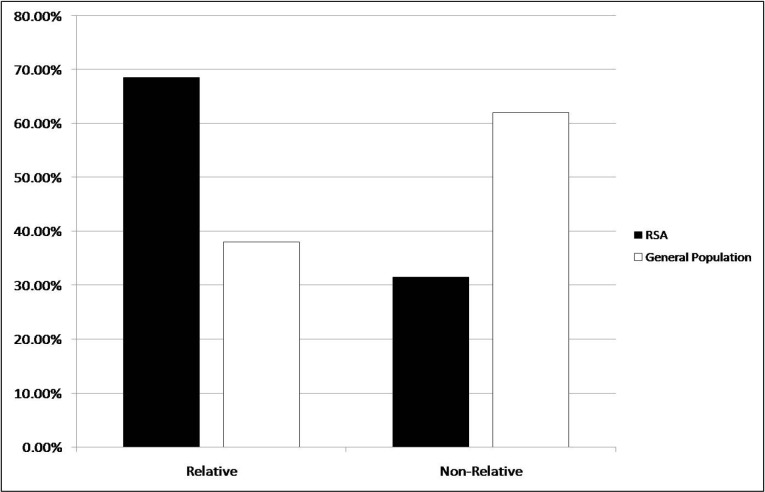
Marriage types in RSA couples

**Fig. 3:**
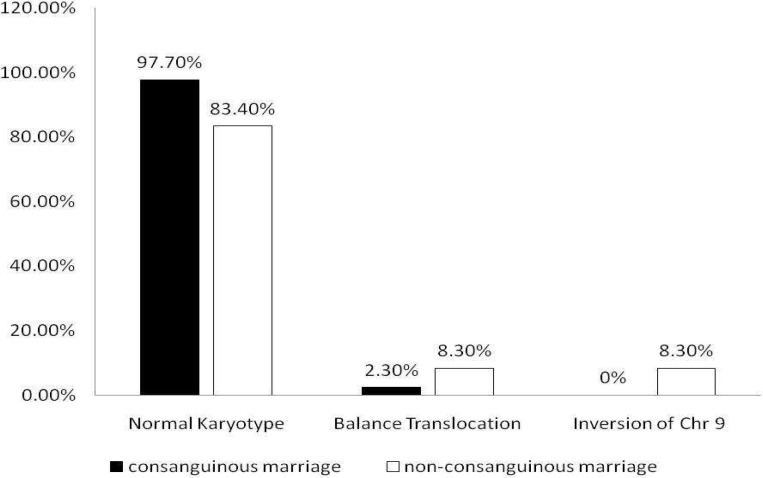
Distribution of balanced chromosomal rearrangements in RSA suffering couples

Age range of recurrent spontaneous abortions (RSA) couples was between 20-39 years with an average of 27.45 years and was not different from general population. The consanguineous marriage rate in RSA couples was compared to 5 years database (from 2007-2012) present in the health center. The comparison was performed using Chi-Square statistical test and results were shown in Figure 2 which indicates that, the consanguineous marriages in blighted ovum suffering couples was significantly (P value <0.001) higher than non-consanguineous marriages (68.5% versus 31.5%), compared to the general population where the major type of marriages was non-consanguineous (62%). Furthermore, results from chromosomal inves-tigation on the basis of G-banding technique

indicated that, the incidences of balanced chromo-somal rearrangements as well as the rate of chromo-some 9 inversion were 8.3 percent each, in non-consanguineous RSA affected couples and the remaining (83.4%) showed apparently normal karyotypes (Fig. 1 and 3). On the other hand balanced chromosomal rearrangements was detected only in 2.3% of RSA affected couples with consanguineous marriage (Fig. 3).

## Discussion

Spontaneous abortion is the most common complication of early pregnancy (1, 18-19). There is a general consensus that healthy woman should not undergo extensive evaluation after single first trimester or early second trimester spontaneous miscarriage which is a relatively common sporadic event. However, miscarriage occurs in about 10 to 15 percent of clinically recognized pregnancies under 20 weeks of gestation (4, 20). It is important to remind that most women with recurrent pregnancy had a good prognosis for eventually having a successful pregnancy even when a definitive diagnosis is not made and no treatment is initiated (5). According to the literature, the prevalence of chromosomal aberrations among couples with repeated spontaneous miscarriages varies in different studies from none to as high as 21.4%. These differences may be related to sample size and to inclusion and exclusion criteria (21-22).

In the present study, couples presenting recurrent spontaneous abortion at the first trimester of gestation with characteristic blighted ovum were candidate for chromosomal investigation since previous studies indicated that abortion occurring at or before 8 weeks of gestation are blighted ovum. From a cytogenetic point of view, previous studies suggested that abnormal fetal karyotypes occur in 90% of unembryonic products of conception (23). This again emphasis that chromosomal aberrations play an important role in blighted ovum presentation. On the other hand, although the incidence of non relative marriages is much higher (62%) than relative marriage (38%) in the studied population, the frequency of recurrent spontaneous abortions (blighted ovum) was much moreevident in consanguineous (68.5%) than non consanguineous marriages (31.5%). Surprisingly, balanced chromosomal rearrangements appeared only in 2.30% of consanguineous marriages compared to non-consanguineous marriages (8.3%) (Fig. 3).

According to the obtained results, the impor-tance of chromosomal studies in non-relative couples affected with recurrent abortion becomes clearer. In this study, Chi square statistical analysis indicated a significant difference between the frequency of RSA occurence between consan-guineous and non-consanguineous marriages (Fig. 2).

Since the structural chromosomal balanced rearrangement are considered as one of the important factors causing recurrent abortion, all couples with RSA were assessed in this study for presence of structural rearrangement.

The results indicated low occurrence of chromosomal abnormalities in this population. The overall frequency of structural abnormalities and reciprocal translocation are approximately 9% and 6% respectively. However, the frequency of structural chromosomal balanced rearrangement is much more in non-consanguineous rather than consanguineous marriages (16.6% versus 2.3%) (Fig. 2). Considering the high prevalence of consanguineous marriages and low prevalence of chromosomal abnormalities with RSA in those marriages, the importance of single gene disorders with autosomal recessive inheritance in the occurrence of RSA becomes evident.

With regard to relatively low incidence of balanced chromosomal rearrangement in consan-guineous couples with blighted ovum, it would be reasonable to suggest that a genetic susceptibitty may play an important role in such pregnancy complications rather than chromosomal disorders. 
